# The Orthopaedic Management of Human Disorganization Syndrome

**DOI:** 10.5435/JAAOSGlobal-D-20-00059

**Published:** 2020-06-15

**Authors:** Kevin Smit, Judy So, Emily Schaeffer, Linlea Armstrong, Cindy Verchere, Kishore Mulpuri

**Affiliations:** From the Division of Orthopedic Surgery, Children's Hospital of Eastern Ontario, Ottawa, ON (Dr. Smit); Faculty of Medicine, University of British Columbia, Vancouver, BC (Ms. So); and Department of Orthopaedics, BC Children's Hospital, Vancouver, BC (Dr. Schaeffer, Dr. Armstrong, Dr. Verchere, and Dr. Mulpuri).

## Abstract

Human disorganization syndrome (HDS) is an extremely rare congenital syndrome characterized by a seemingly random distribution of multiple developmental anomalies involving all three germinal layers.

**Case Report:**

We report a rare case of a female child whose congenital anomalies are consistent with HDS. The orthopaedic features of this patient include a popliteus pterygium with an associated flexion contracture secondary to an elongated biceps femoris tendon that attached to the gastrocnemius-soleus muscle complex, two finger-like appendages, a tethered cord, a lipomeningomyelocele at the level of L5, and a leglength discrepancy. The patient was treated with a splinting program, release of the biceps femoris tendon at its erroneous insertion from the gastrocs-soleus, and surgical excision of the finger-like appendages. She underwent three subsequent soft-tissue releases to address recurrence of the knee flexion contracture and an anteromedial and lateral distal femoral eight plate procedure for guided growth and slow correction of the remaining flexion deformity.

**Conclusion:**

The treatment of HDS can be quite complex and can present with a variety of anomalies with distinctive orthopaedic features correctable with surgical management, including soft-tissue releases, excision of appendages, and growth modulation.

Disorganization syndrome (DS) was first described in 1958 by Hummel^1^ when he observed a mutant mouse with a disruption of the orderly process of organogenesis, resulting in a seemingly random distribution of independent anomalies derived from all three germ layers. The wide range of malformations observed in DS poses a challenge in outlining the diagnostic criteria. The most commonly reported malformations include the following: cranioschisis/exencephaly, unusual hamartomas, appendicular anomalies, eye malformations, pharyngeal defects, and gastroschisis.^1,2^ Extremity abnormalities include duplications and reductions, usually involving a single limb, polydactyly, and malformed and small limb girdles.^1^

In 1989, Donnai and Winter^3^ proposed a human homologue of DS and described the case of an infant with multiple defects in-line with those observed in DS mice. The infant presented with shortening of the right leg, a web across the popliteus fossa, nine toes on one foot, a finger-like structure arising from the abdomen, and an absent kidney. Since then, few case reports have been published on patients suspected to have Human DS (HDS).^4,5^

Current case reports have almost exclusively focused on medical genetics despite many HDS-associated anomalies requiring orthopaedic intervention. Here, we report the orthopaedic management of a patient presenting with congenital physical anomalies consistent with HDS.

## Case Report

This report describes a female child who was delivered at term via elective caesarean section to healthy, nonconsanguineous parents after an uncomplicated pregnancy. The patient also has an older female sibling who displays no abnormalities. Prenatal chromosomal studies were negative for fetal abnormalities; however, a number of congenital anomalies were observed at birth.

**Figure 1:**
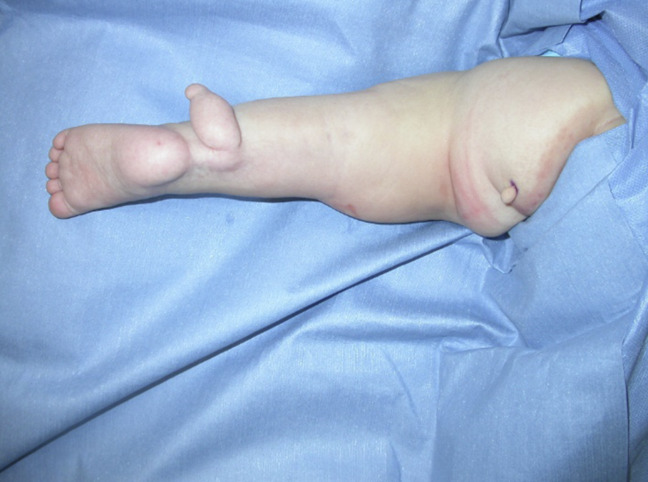
An intra-operative image showing two supernumerary appendages.

The anomalies included a left popliteus pterygium extending from the buttock to the calcaneus, with an associated knee flexion contracture. In addition, a toe-like skin tag was observed on the posterior aspect of the calf and another smaller skin tag on the lower left aspect of the buttock; it appeared that the two appendages had previously been connected (Figure 1). A cutaneous capillary malformation (port-wine-stain) in the right sacral region was also observed. Ultrasonography was used to identify an underlying terminal L5 lipomeningomyelocele with tethered cord and left uretral duplication with hydronephrosis and an ureterocele. The spinal abnormalities were appropriately monitored by neurosurgery, and the renal anomaly was treated with a left upper pole heminephrectomy by urology.

**Figure 2:**
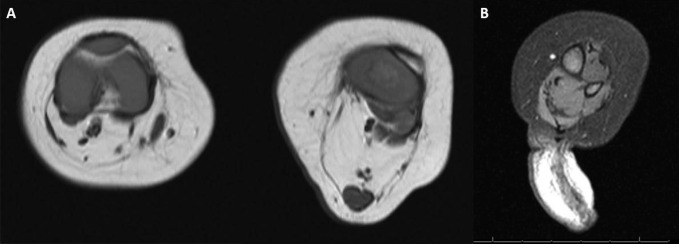
**A**, MRI of the left leg demonstrating the source of the pterygium to be a long biceps femoris tendon. **B**, MRI of left leg showing the toe-like skin tag on the posterior aspect of the calf.

The popliteus pterygium caused a 40° knee flexion contracture that was slightly flexible. A full-time splinting program was initiated, and at 3 months of age, her passive range improved to full extension, facilitating subsequent weaning to night-time splinting. An MRI of the left leg revealed that a long biceps femoris tendon was the source of the pterygium. It extended from the ischium to calcaneus and connected the biceps femoris muscle to the gastrocnemius-soleus muscle complex (Figure 2). Owing to the recurrence of her knee contracture after splinting, a decision was made to proceed with surgical intervention.

**Figure 3:**
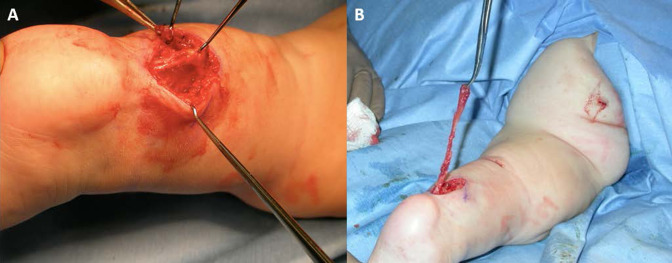
**A**, An intraoperative image showing the posterior approach centered over the Achilles tendon taken to correct the pterygium. **B**, An intra-operative image showing the long biceps femoris tendon before excision.

At nine months of age, excision of the distal 5.5 cm of the biceps femoris tendon off of the calcaneus was performed through a posterior approach centered over the Achilles tendon (Figure 3, A). A proximal transverse incision at the knee crease was made to identify the tendon proximally (Figure 3, B). During this surgical procedure, the two accessory appendages were also surgically excised. The large finger-like skin tag, associated with the left leg pterygium, was found in the muscle belly of the left calf at the proximal end of the Achilles tendon. The smaller skin tag was found to arise from the lower left buttock with no significant attachments.

**Figure 4:**
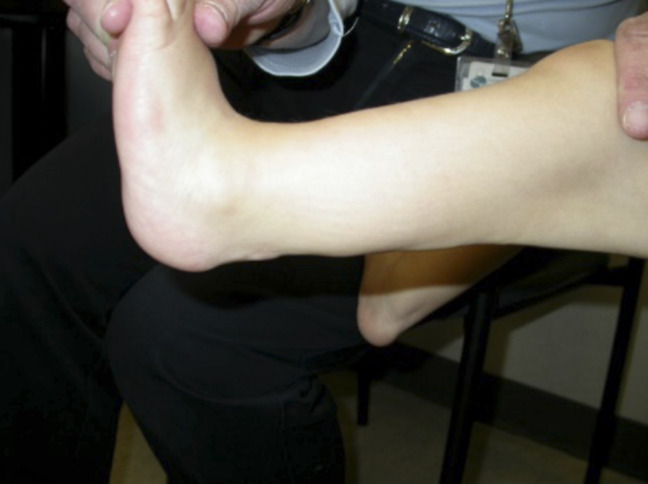
Six-month postoperative image of left leg showing regrowth of the fibrous band behind her knee.

Six months postoperatively, the patient had recurrence of a 15° knee flexion contracture despite aggressive stretching and bracing treatment. Regrowth of the fibrous band behind her knee was noted at this time (Figure 4). Consequently, excision of remnant lateral fibers and release of her medial hamstring tendons were performed through a longitudinal incision proximal to the posterior knee crease. The patient had near full extension intraoperatively; however, a tight neurovascular bundle was identified and precluded further release to maintain the neurovascular status of the lower extremity.

**Figure 5:**
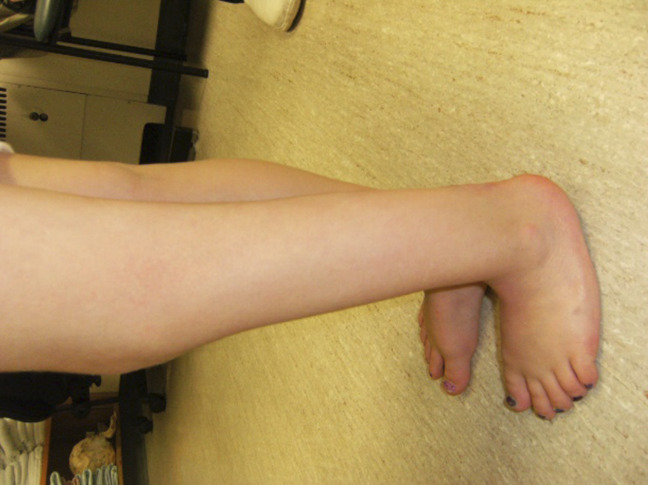
A postoperative image after the patient's second orthopaedic procedure. Her left knee was only 5° short of full extension at this time.

By the age of five, the knee flexion contracture had increased to 30°, and the patient also presented with a 20° equinus contracture of her foot and a 1.5-cm leg length discrepancy. A gastroc recession, hamstring lengthening, and distal tendoachilles lengthening was done. Her postoperative rehabilitation course involved wearing an articulated ankle-foot orthosis during the day and a knee-ankle-foot orthosis at night. After the operation and rehab course, her knee was only 5° short of full extension and she had 10° ankle dorsiflexion and 40° plantar flexion (Figure 5). The 1.5-cm leg length discrepancy remained stable from age five to seven.

**Figure 6:**
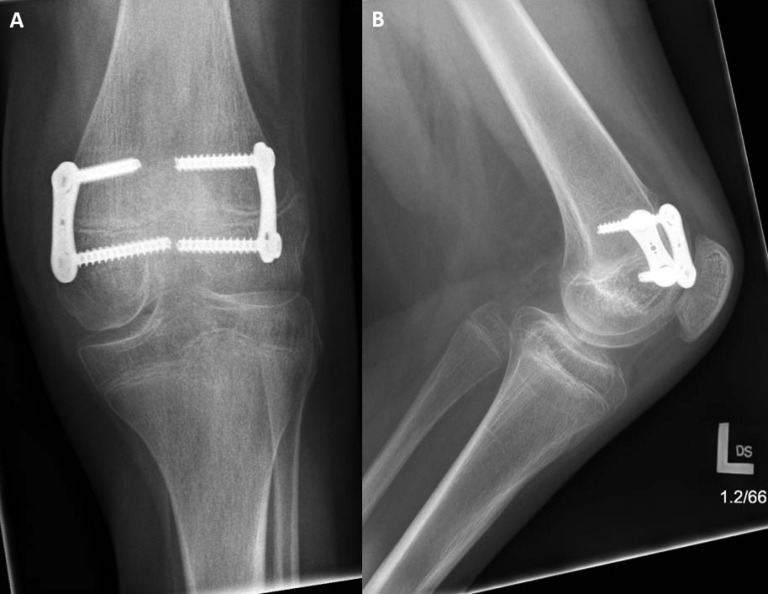
**A**, An AP radiograph of the patient's left knee demonstrating placement eight-plates postoperatively. **B**, A lateral radiograph of the patient's left knee, showing the eight plates from a different angle.

At age eight, she redeveloped a 15° to 20° fixed flexion deformity, and a revision hamstring release was done. A year after this procedure, additional procedures to lengthen the hamstring and Achilles tendons were done because of increased knee flexion deformity. Immediately after these procedures, her knee was straight and her ankle remained in the neutral position for 3 years, before she redeveloped a 25° left knee flexion contracture at age 12. An anteromedial and lateral distal femoral eight plate–guided growth procedure was done to allow for slow correction of this flexion deformity. Unfortunately, because of failure of the proximal screw and associated pain, her knee flexion contracture worsened to 40°. A revision surgery was done four months after the initial procedure to replace the plate, and her knee flexion contracture decreased to 25° (Figure 6).

Her most recent visit to the orthopaedic clinic occurred five months after her last procedure, and her knee flexion contracture remained at 25°. It was also noted during this visit that she was losing flexion in her hips. The patient is 14 as of 2020 and will be monitored closely into adulthood in case any further changes occur.

## Discussion

DS was first described and characterized in laboratory mice with a severe pattern of malformations in several body parts, many of which seemed to derive from the three germ layers.^1^ Based on the existing case reports, patients presenting with a bizarre constellation of malformations that cannot be explained by a single syndrome should be considered for a diagnosis of HDS.^6,7^

In the past 25 years, limited reported cases of HDS were noted because the reported frequency is less than 1:2,600,000 newborns.^8^ The physical manifestations seen in our patient are consistent with previously published reports of HDS. Although no two reported cases of HDS exhibit identical combinations of anomalies, recurring features noted in the literature were observed in this case, including digit-like appendages,^3,6,9,10,11,12,13,14,15,16,17,18,19,20,21,22^ popliteus pterygia and/or knee flexion deformities,^6,11-13,18,23,24^ and renal defects.^3,6,11-13,16,18,20,23,24^ The patient also exhibited normal intellectual development, consistent with other reports.^9,12,15,17,20,23^

Previous published reports of HDS are largely focused on the pathoanatomy and medical genetics of HDS. It is thought that the HDS phenotype is because of a semidominant mutation with incomplete penetrance, which is usually lethal in the homozygous state.^1,2^ The low penetrance and high variability of clinical manifestations of HDS make it a paradigm for understanding. A “two-hit” hypothesis has been proposed to explain the variability in presentation of HDS, where the first hit is the genetic mutation and the second hit is an epigenetic or external event.^25^ To date, the gene(s) for HDS remains unknown.

This case highlighted the presence of a popliteus pterygium secondary to an elongated biceps femoris tendon that attached to the gastrocnemius-soleus muscle complex. This resulted in a knee flexion contracture that proved difficult to treat with both nonoperative splinting and surgical resection. Popliteus pterygium has been described as one of the most difficult orthopaedic problems.^26^ The indications for the treatment of popliteus pterygium have been functional limitations in ambulation and deformity. Many surgical interventions have been described, including a variety of soft-tissue releases^26,27,28,29^ and bony osteotomies.^26,30,31^ The sciatic nerve is often a limiting factor in complete lengthening of the skin and soft tissues in the affected area because the nerves and vessels are shortened. In addition, the sciatic nerve may be attached to the fibrous band of anomalous muscle, known as the calcaneo ischiadicus.^32,33^ Recurrence of the flexion contracture is frequent, and repeat surgical correction is common, as demonstrated in the present case.^26^

## Conclusion

HDS is a complex condition with distinctive orthopaedic features that are at least partly correctable with surgical management. In this case, several orthopaedic surgical interventions were done because of the recurrence of flexion contractures. Further case reports of HDS and discussion in the orthopaedic community to identify similar cases are required to learn more about this extremely rare disorder. This may allow for the identification of possible risk factors and aid accurate diagnosis and management of HDS-related orthopaedic conditions in the future.

## References

[1] Hummel KP: The inheritance and expression of disorganization, an unusual mutation in the mouse. J Exp Zool 1958;137:389-423. 13587873 10.1002/jez.1401370303

[2] Hummel KP: Developmental anomalies in mice resulting from action of the gene, disorganization, a semi-dominant lethal. Pediatrics 1959;23:212-221. 13613883

[3] Donnai D Winter RM: Disorganization: A model for “early amnion rupture”? J Med Genet 1989;26:421-425. 2746613 10.1136/jmg.26.7.421 PMC1015643

[4] Hunter AG: Human equivalent of mouse disorganization: Has the case been made? Am J Med Genet A 2011;155A:792-804. 21416595 10.1002/ajmg.a.33910

[5] Vallejo OG Sanchez M Canovas CS Ontiveros JD: Patient with disorganization syndrome: Surgical procedures, pathology, and potential causes. Birth Defects Res A Clin Mol Teratol 2013;97:781-785. 24307594 10.1002/bdra.23203

[6] Naguib KK Hamoud MS Khalil ES El-Khlalifa MY: Human homologue for the mouse mutant disorganization: Does it exist? J Med Genet 1991;28:138-139. 2002487 10.1136/jmg.28.2.138 PMC1016786

[7] Teebi AS Elliott AM: Another case of the human homologue of the mouse mutant disorganization. Am J Med Genet 1996;61:94. 8741929 10.1002/ajmg.1320610106

[8] Wax JR Pinette MG Smith R Cartin A: Prenatal sonographic findings in human disorganization syndrome. J Ultrasound Med 2010;29:301-305. 20103803

[9] Gonzalez de Dios J Bermejo E Mestre J: Disorganization syndrome: Characteristics and description of the first case registered by ECEMC. Bol ECEMC Rev Dismor Epidemiol 2010;5:9-14.

[10] De Michelena MI Stachurska A: Multiple anomalies possibly caused by a human homologue to the mouse disorganization (Ds) gene. Clin Dysmorphol 1993;2:131-134. 8281274

[11] Elliott AM Chen MF Azouz EM Teebi AS: Developmental anomalies suggestive of the human homologue of the mouse mutant disorganization. Am J Med Genet 1995;55:240-243. 7717426 10.1002/ajmg.1320550218

[12] Mallory SB Krafchik BR Hivnor CM Yan AC: What syndrome is this? Disorganization syndrome. Pediatr Dermatol 2007;24:90-92. 17300661 10.1111/j.1525-1470.2007.00344.x

[13] Kabra M Suri M Jain U Verma IC: Poland anomaly with unusual associated anomalies: Case report of an apparent disorganized defect. Am J Med Genet 1993;52:402-405. 10.1002/ajmg.1320520403 7747752

[14] Korniszewski L Skorka A Donnai D: Disorganization: A case with popliteal pterygia and placental-skin appendages. Clin Dysmorphol 1999;8:277-281. 10532177

[15] Lin AE: Two additional patients representing the possible human homologue for the mouse mutant disorganization (Ds). J Med Genet 1991;28:645-647. 1956071 10.1136/jmg.28.9.645 PMC1015804

[16] Lowry RB Yong SL: Cleft lip and palate, sensorineural deafness, and sacral lipoma in two brothers: A possible example of the disorganization mutant. J Med Genet 1991;28:135-137. 2002486 10.1136/jmg.28.2.135 PMC1016785

[17] O'Driscoll MC Peckham C Kerr B: Four limb syndactyly, constriction of rings and skin tags; amniotic bands or disorganization syndrome. Clin Dysmorphol 2008;17:255-258. 18978653 10.1097/MCD.0b013e328310e07d

[18] Onal EE Canan T Yldz A Nurullah O: Tubular skin appendage, renal agenesis and popliteal web: A further example of the human homologue of disorganization (Ds). Clin Dysmorphol 2005;14:89-91. 15770131

[19] Petzel MA Erickson RP: Disorganization: A possible cause of apparent conjoint twinning. J Med Genet 1991;28:712-714. 1941969 10.1136/jmg.28.10.712 PMC1017061

[20] Robin NH Adewale OO McDonald-McGinn D Nadeau JH: Human malformations similar to those in the mouse mutation disorganization (Ds). Hum Genet 1993;92:461-464. 8244336 10.1007/BF00216451

[21] Stanek J de Courten-Myers G Spaulding AG Strub W: Case of complex craniofacial anomalies, bilateral nasal proboscides, palatal pituitary, upper limbs reduction, and amnion rupture sequence: Disorganization phenotype? Pediatr Dev Pathol 2001;4:192-202. 11178637 10.1007/s100240010131

[22] van Langen IM Hennekam RC: Another example of the human homologue of the mouse mutant disorganization? Clin Dysmorphol 1994;3:361-362. 7894744

[23] Hennekam RC: Another human homologue for the mouse mutant disorganisation. J Med Genet 1992;29:71. 10.1136/jmg.29.1.71-b PMC1015836 1552552

[24] Winter RM Donnai D: A possible human homologue for the mouse mutant disorganization. J Med Genet 1989;26:417-420. 2664177 10.1136/jmg.26.7.417 PMC1015642

[25] Crosby JL Varnum DS Nadeau JH: Two-hit model for sporadic congenital anomalies in mice with the disorganization mutation. Am J Hum Genet 1993;52:866-874. 8488837 PMC1682043

[26] Parikh SN Crawford AH: Popliteal pterygium syndrome: Implications for orthopaedic management. J Pediatr Orthop B 2004;13:197-201. 15083121 10.1097/00009957-200405000-00010

[27] Gardetto A Piza-Katzer H: A case of familial popliteal pterygium syndrome: Early surgical intervention for successful treatment. Pediatr Surg Int 2003;19:612-614. 12961094 10.1007/s00383-003-1062-x

[28] Solignac N Vialle R Thevenin-Lemoine C Damsin JP: Popliteal pterygium knee contracture: Treatment with the Ilizarov technique. Orthop Traumatol Surg Res 2009;95:196-201. 19410531 10.1016/j.otsr.2009.01.004

[29] Tuerk D Edgerton MT: The surgical treatment of the congenital webbing (pterygium) of the popliteal area. Plast Reconstr Surg 1975;56:339-444. 1153550 10.1097/00006534-197509000-00020

[30] Lampasi M Antonioli D Donzelli O: Management of knee deformities in children with arthrogryposis. Musculoskelet Surg 2012;96:161-169. 22875688 10.1007/s12306-012-0218-z

[31] Saleh M Gibson MF Sharrard WJ: Femoral shortening in correction of congenital knee flexion deformity with popliteal webbing. J Pediatr Orthop 1989;9:609-611. 2794038 10.1097/01241398-198909010-00020

[32] Addison A Webb PJ: Flexion contractures of the knee associated with popliteal webbing. J Pediatr Orthop 1983;3:376-379. 6874938 10.1097/01241398-198307000-00019

[33] Porat S Mosheiff R Peyser A: Popliteal pterygium associated with complete amelia of upper limb: Early surgical treatment. J Pediatr Orthop 1995;15:254-259. 7745103

